# Proteasome-mediated degradation of keratins 7, 8, 17 and 18 by mutant KLHL24 in a foetal keratinocyte model: Novel insight in congenital skin defects and fragility of epidermolysis bullosa simplex with cardiomyopathy

**DOI:** 10.1093/hmg/ddab318

**Published:** 2021-11-05

**Authors:** Elena Logli, Elisa Marzuolo, Marco D’Agostino, Libenzio Adrian Conti, Anna Maria Lena, Andrea Diociaiuti, Elena Dellambra, Cristina Has, Valentina Cianfanelli, Giovanna Zambruno, May El Hachem, Alessandra Magenta, Eleonora Candi, Angelo Giuseppe Condorelli

**Affiliations:** Genodermatosis Unit, Genetics and Rare Diseases Research Division, Bambino Gesù Children’s Hospital, IRCCS, Piazza Sant’Onofrio 4, 00165, Rome, Italy; Genodermatosis Unit, Genetics and Rare Diseases Research Division, Bambino Gesù Children’s Hospital, IRCCS, Piazza Sant’Onofrio 4, 00165, Rome, Italy; Laboratory of Experimental Immunology, IDI-IRCCS, Via Monti di Creta 104, 00167, Rome, Italy; Confocal Microscopy Core Facility, Bambino Gesù Children’s Hospital, IRCCS, Viale di San Paolo 15, 00146, Rome, Italy; Department of Experimental Medicine, University of Rome “Tor Vergata”, Via Montpellier 1, 00133, Rome, Italy; Dermatology Unit and Genodermatosis Unit, Genetics and Rare Diseases Research Division, Bambino Gesù Children’s Hospital, IRCCS, Piazza Sant’Onofrio 4, 00165, Rome, Italy; IDI-IRCCS, Via Monti di Creta 104, 00167, Rome, Italy; Department of Dermatology, Medical Faculty, Medical Center – University of Freiburg, Freiburg, Germany; Department of Pediatric Hemato-Oncology and Cell and Gene Therapy, Bambino Gesù Children’s Hospital, IRCCS, Piazza Sant’Onofrio 4, 00165, Rome, Italy; Genodermatosis Unit, Genetics and Rare Diseases Research Division, Bambino Gesù Children’s Hospital, IRCCS, Piazza Sant’Onofrio 4, 00165, Rome, Italy; Dermatology Unit and Genodermatosis Unit, Genetics and Rare Diseases Research Division, Bambino Gesù Children’s Hospital, IRCCS, Piazza Sant’Onofrio 4, 00165, Rome, Italy; Institute of Translational Pharmacology (IFT), National Research Council of Italy (CNR), Via Fosso del Cavaliere 100, 00133, Rome, Italy; Department of Experimental Medicine, University of Rome “Tor Vergata”, Via Montpellier 1, 00133, Rome, Italy; IDI-IRCCS, Via Monti di Creta 104, 00167, Rome, Italy; Genodermatosis Unit, Genetics and Rare Diseases Research Division, Bambino Gesù Children’s Hospital, IRCCS, Piazza Sant’Onofrio 4, 00165, Rome, Italy

## Abstract

Epidermolysis bullosa simplex (EBS) with cardiomyopathy (EBS-KLHL24) is an EBS subtype caused by dominantly inherited, gain-of-function mutations in the gene encoding for the ubiquitin-ligase KLHL24, which addresses specific proteins to proteasomal degradation. EBS-KLHL24 patients are born with extensive denuded skin areas and skin fragility. Whilst skin fragility rapidly ameliorates, atrophy and scarring develop over time, accompanied by life-threatening cardiomyopathy. To date, pathogenetic mechanisms underlying such a unique disease phenotype are not fully characterized. The basal keratin 14 (K14) has been indicated as a KLHL24 substrate in keratinocytes. However, EBS-KLHL24 pathobiology cannot be determined by the mutation-enhanced disruption of K14 alone, as K14 is similarly expressed in foetal and postnatal epidermis and its protein levels are preserved both *in vivo* and *in vitro* disease models. In this study, we focused on foetal keratins as additional KLHL24 substrates. We showed that K7, K8, K17 and K18 protein levels are markedly reduced *via* proteasome degradation in normal foetal keratinocytes transduced with the mutant KLHL24 protein (ΔN28-KLHL24) as compared to control cells expressing the wild-type form. In addition, heat stress led to keratin network defects and decreased resilience in ΔN28-KLHL24 cells. The KLHL24-mediated degradation of foetal keratins could contribute to congenital skin defects in EBS-KLHL24. Furthermore, we observed that primary keratinocytes from EBS-KLHL24 patients undergo accelerated clonal conversion with reduced colony forming efficiency (CFE) and early replicative senescence. Finally, our findings pointed out a reduced CFE in ΔN28-KLHL24-transduced foetal keratinocytes as compared to controls, suggesting that mutant KLHL24 contributes to patients’ keratinocyte clonogenicity impairment.

## Introduction

Kelch-like (KLHL) protein family encompasses a large, evolutionarily conserved and heterogeneous group of adaptor molecules generally involved in the ubiquitination process (1). KLHL proteins (KLHLs) mainly work as intermediaries between the cullin 3 (Cul3) ubiquitin ligase and its substrates, which vary from cytoskeleton intermediate filaments to transcription factors or specific serine/threonine kinases depending on the KLHL family member and cellular context (1–8). KLHLs affect a multitude of ubiquitin-mediated cellular functions including cytoskeleton component degradation (9–11), mitosis progression (4–6), autophagy (12–14), and oxidative stress response (8). In keeping with the pervasive role of ubiquitination in biological processes, aberrations in KLHL protein functioning underlie different pathological conditions, including inherited diseases (9,10,15–18).

Epidermolysis bullosa simplex (EBS) is a clinically and genetically heterogeneous disorder characterized by skin fragility and blister formation within the basal layer of the epidermis (19,20). The commonest subtypes of EBS are due to dominant mutations in *KRT5* and *KRT14* genes encoding for the basal keratin 5 (K5) and K14, respectively (19,20). Monoallelic mutations in the translation initiation codon of the *Kelch-like family member 24* (*KLHL24*) gene are causative of a recently discovered autosomal dominant syndromic EBS subtype, EBS intermediate with cardiomyopathy, hereafter indicated as EBS-KLHL24 (15,16). EBS-KLHL24 has peculiar skin phenotypic features: patients are always born with large denuded skin areas, which heal with marked atrophic scarring. Skin fragility rapidly ameliorates already in infancy (15,16,21,22). In addition, EBS-KLHL24 patients develop in the early adulthood a life-threatening dilated cardiomyopathy determined by the KLHL24-mediated degradation of the intermediate filament desmin in cardiomyocytes (22–27). Conversely, loss of function mutations in KLHL24 are associated with increased desmin expression in cardiac and skeletal muscle tissues of patients affected with hypertrophic cardiomyopathy (17). Taken together, these evidences highlight the crucial KLHL24 role also in heart physiopathology.

All *KLHL24* monoallelic mutations identified to date in EBS-KLHL24 patients result in a truncated protein, named ΔN28-KLHL24, missing the first 28 amino acids at the N-terminal end. KLHL24 contains autoubiquitination sites involved in modulating protein levels through proteasomal degradation (16). The mutant ΔN28-KLHL24 lacks at least one autoubiquitination site, leading to increased stability and biological activity as compared to the wild-type protein form (16). Although to date K14 represents the main KLHL24 target in keratinocytes, discordant evidence has emerged from both *in vitro* mechanistic studies and *in vivo* findings in EBS-KLHL24 patients (11,15,16). Lin and coll. Reported that K14 was strongly reduced in skin whole tissue lysates from biopsies collected at the margin of full-blown blisters of two EBS-KLHL24 children (see Supplementary Figure 2 in ref. (16)) as compared to healthy controls. Similar findings were obtained in *Klhl24*^c.3G/T^ mice which, however, did not manifest skin fragility (16). Conversely, we and others could not detect significant differences in K14 expression levels between EBS-KLHL24 patients and controls both *in vivo* and *in vitro* (13,15,22,23). Thus, the target(s) and function of KLHL24 in the skin remain elusive.

The aims of the present study were to molecularly characterize the clonogenic and self-renewal properties of primary keratinocytes from EBS-KLHL24 patients and to search for targets of KLHL24 during foetal epidermal development. We show that primary EBS-KLHL24 undergo rapid clonal conversion and premature senescence, and identify for the first time a set of keratins (*i.e.* keratin 7, 8, 17 and 18) expressed during foetal development (28–31) that undergo proteasome-mediated degradation in ΔN28-KLHL24-transduced primary foetal keratinocytes. Altogether, our findings contribute to decipher the molecular bases underlying the unique skin phenotype of EBS-KLHL24 disease.

## Results

### Comparable keratin 14 protein levels in the skin and primary keratinocytes of EBS-KLHL24 patients and controls

**Figure 1:**
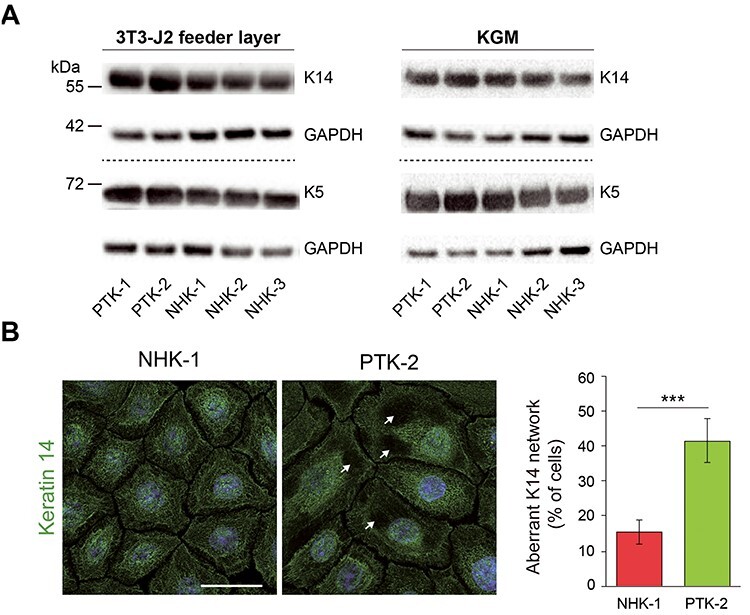
Primary EBS-KLHL24 patient keratinocytes display preserved expression levels of basal keratins 5 and 14, but altered organization of keratin 14. (**A**) Immunoblot (IB) showing keratin 14 (K14) and K5 protein levels in primary keratinocytes from EBS-KLHL24 patients (PTK-1 and -2) and age-matched healthy controls (NHK-1, −2 and − 3) grown on a feeder layer of 3T3-J2 fibroblasts in DMEM/F12 supplemented with 10% foetal calf serum (**left panel**) or cultured in defined medium (KGM, Keratinocyte Growth Medium) (**right panel**). Glyceraldheyde-3-phosphate dehydrogenase (GAPDH) was used as loading control. Dotted lines separate distinct IB experiments. (**B, left panel**) Representative confocal z-stack images of NHK-1 and PTK-2 stained with K14 antibody (green). Nuclei were stained with Hoechst 33258 (blue). Note the presence of focal areas of reduced K14 cytoplasmic staining in PTK-2 (white arrows) suggestive of an aberrant K14 network in patient keratinocytes. Scale bar = 30 μm. (**B, right panel**) Histogram showing the percentage of keratinocytes (NHK-1 and PTK-2) hallmarked by an aberrant K14 staining pattern. Values were calculated using the following formula: (number of cells presenting focal areas of reduced K14 staining x 100/the total number of cells in each field). A minimum of four fields acquired with a 40X objective were analysed, corresponding to a total count of 236 NHKs and 146 PTKs. Data are presented as mean ± SD. ^*^^*^^*^P < 0.001.

Primary keratinocytes were established from perilesional skin biopsies of two EBS-KLKL24 children (patient 1 and 2, PT-1 and PT-2). PT-1 carries the *de novo* heterozygous c.2 T > C (p.Met1?) mutation in *KLHL24* gene (Case 1, (22)), and the same *de novo* KLHL24 mutation was identified in PT-2 who was born with extensive aplasia cutis of upper and lower limbs healing with skin atrophy and scarring (Supplementary Material, Fig. S1). Immunofluorescence antigen mapping of PT-2 skin showed cleavage within the basal epidermal layer together with normal expression of major keratinocyte adhesion proteins, including K14 and K5 (not shown). Similar findings have been described for PT-1 (22). In keeping with our *in vivo* results, expression of K14 and K5 in patient keratinocytes (PTKs) was similar to three normal human keratinocyte (NHK) strains grown on 3T3-J2 fibroblast feeder layer in DMEM/F12 supplemented with 10% foetal calf serum or in KGM (Keratinocyte Growth Medium) defined medium, in the absence of feeder layer (Fig. 1A). However, confocal microscopy analysis pointed out a disorganization of K14 network with focal areas of reduced K14 cytoplasmic staining in PTKs grown in KGM (Fig. 1B). Consistently with previous findings (15,21–23), our results show that K14 expression levels are preserved in the skin and primary keratinocytes of EBS-KLHL24 children, although K14 network disorganization was observed.

### Reduced lifespan and accelerated clonal conversion in EBS-KLHL24 primary keratinocytes

EBS-KLHL24 children present progressive skin atrophy, a feature of elderly individuals. Normal human epidermis undergoes continual and rapid self-renewal, a process that relies on stem and transient amplifying cells. Clonal conversion from stem to transient amplifying cells takes place during natural skin aging as well as keratinocyte sub-cultivation (32,33). *In vitro* keratinocyte replicative senescence occurs when clonal evolution is completed and transient amplifying cells have exhausted their proliferative potential, entered terminal differentiation and generated paraclones (*i.e.* aborted colonies constituted only by terminally differentiated cells). Thus, aborted colony percentage is a direct measure of clonal conversion accomplishment and stem cell depletion.

**Figure 2:**
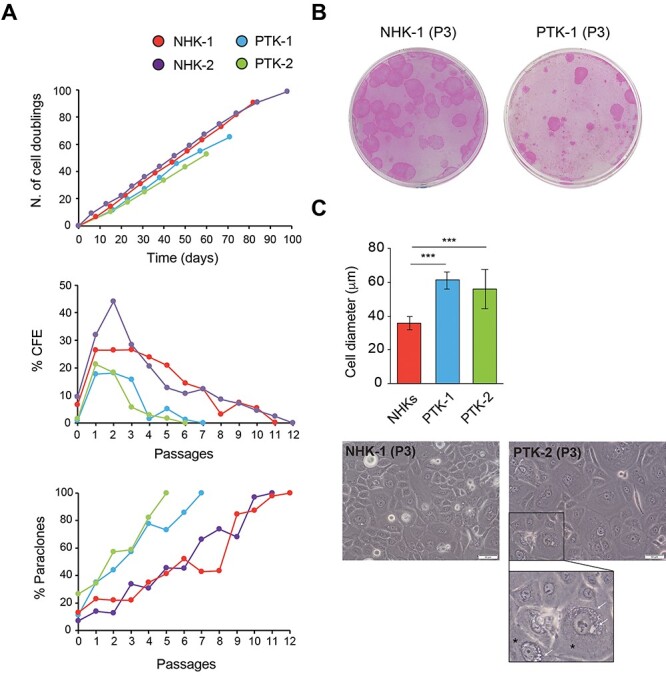
Clonal analysis of primary keratinocytes from EBS-KLHL24 patients reveals reduced lifespan, increased paraclone percentage and altered cell morphology. (**A, upper panel**) The graph shows the cumulative number of cell doublings per passage (y axis) of primary keratinocytes from EBS-KLHL24 patients (PTKs, PTK-1 and -2) and healthy controls (NHKs, NHK-1 and -2), plotted against the number of days in culture (x axis). The number of cell doublings was calculated using the following formula: x = 3.322 log N/No., where N is the total number of cells obtained at each passage and No. is the number of clonogenic cells, calculated from colony forming efficiency (CFE) data. (**A, middle panel**) CFE of PTKs and NHKs at different passages. The percentage of CFE was evaluated in parallel at each passage and expressed as the ratio of the number of colonies on the number of inoculated cells, and then plotted against the cell passages. (**A, lower panel**) The graph illustrates the percentage of aborted colonies (paraclones) in PTKs and NHKs. Values are expressed as the ratio between the number of aborted colonies and the total number of colonies, plotted against the cell passages. (**B**) Representative images of NHK-1 and PTK-1 colonies at passage 3 (P3). (**C, upper panel**) Histogram showing the average diameter of PTK-1, PTK-2 and two NHK strains grown in defined medium until P3. Cells were photographed in bright-field with an Olympus iX71 inverted microscope, and analysed using ImageJ software (NIH, Bethesda, MD, USA). The keratinocyte mayor axis length was measured to obtain cell diameter values. Data are presented as mean ± SD of the diameter of keratinocyte strains. A total of 371 NHKs and 406 PTKs were counted. ^*^^*^^*^P < 0.001. (**C, lower panel**) Representative images showing NHK-1 and PTK-2 grown in defined medium, at P3. The inset (lower panel) shows a higher magnification of two large keratinocytes with abundant cytoplasm (asterisks) presenting numerous vacuole-like perinuclear structures of different size (white arrows). Scale bar = 50 μm.

As the clonogenic and self-renewal properties of PTKs have not been investigated to date, primary keratinocytes from two genotyped EBS-KLHL24 patients and two age-matched control subjects (NHK-1 and NHK-2) were serially cultivated on 3T3-J2 fibroblast feeder-layer. In parallel, colony forming efficiency (CFE) and percentage of paraclones were measured at each culture passage. PTKs showed a reduced lifespan, in the range of 52–65 cell doublings, compared to the average value of 94 doublings of NHKs (Fig. 2A, upper panel). PTK CFE values were lower than NHK at passage 0 (P0), *i.e.* cells seeded after isolation from skin biopsy (1.2% as average value in PTKs *versus* 8% in NHKs) (Fig. 2A, middle panel). During subcultivation, clonogenicity of PTKs and NHKs further diverged (Fig. 2A, middle panel and Fig. 2B). In line with CFE findings, PTKs showed a higher percentage of abortive colonies already at early passages (*e.g.* 34.6% as average value of PTKs *versus* 18.5% of NHKs at P1) (Fig. 2A, lower panel). Accordingly, the proliferation rate of PTKs grown in KGM proved significantly lower than that of NHKs (Supplementary Material, Fig. S2A). In addition, low passage PTKs cultured in defined medium presented a senescent-like morphology, hallmarked by greater cell dimensions as compared to NHKs (Fig. 2C, upper panel) and vacuole-like structures of different size frequently observed in a perinuclear localization (Fig. 2C, lower panel). Taken together, these findings pointed to a premature senescence of PTKs.

### Premature replicative senescence in EBS-KLHL24 primary keratinocytes

**Figure 3:**
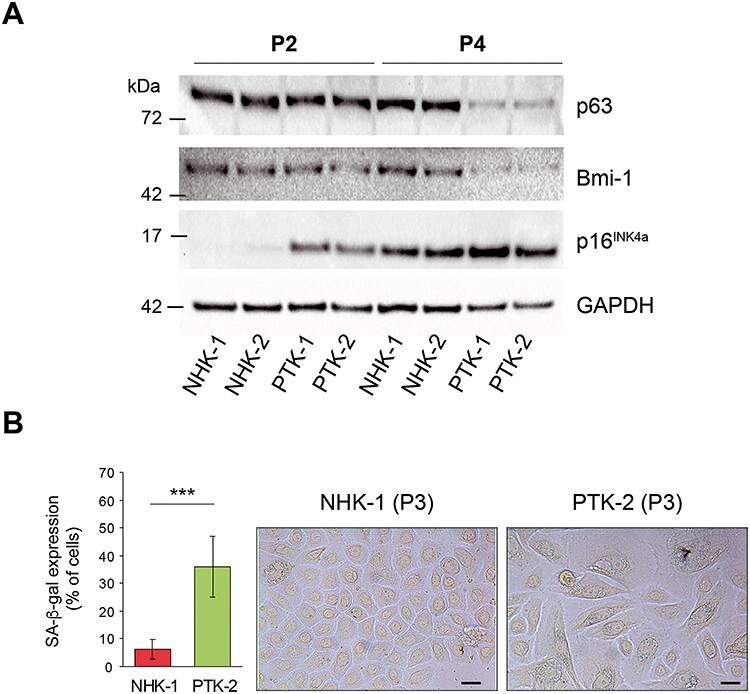
Primary keratinocytes from EBS-KLHL24 patients display premature senescence features. (**A**) Immunoblot (IB) showing p63, Bmi-1 and p16^INK4a^ protein levels in primary keratinocytes from EBS-KLHL24 patients (PTK-1 and -2) and healthy controls (NHK-1 and -2) grown in defined medium, at passage 2 (P2) and P4. Image Lab Software (Bio-Rad) was employed to quantify protein expression levels. Glyceraldheyde-3-phosphate dehydrogenase (GAPDH) was used as loading control. (**B, left panel**) Histogram showing the percentage of positive cells for the senescence-associated ß-galactosidase (SA-ß-gal) activity in NHK-1 and PTK-2. Values are expressed as the ratio between the number of cells with blue staining and the total number of cells counted in each microscopy field. A total of 356 NHKs and 412 PTKs were counted. ^*^^*^^*^P < 0.001. (**B, right panel**) Representative images of NHK-1 and PTK-2, keratinocytes showing SA-ß-gal activity are stained with blue-colour dots. Scale bar = 25 μm.

In aged keratinocyte cultures, the paraclone percentage increase correlates with the up-regulation of p16^INK4a^, a replicative senescence marker (34), and the down-regulation of p63 and Bmi-1, two proteins typifying keratinocytes endowed with high proliferative capacity (33,35,36). In order to evaluate the accumulation of senescent cells during time, we investigated p16^INK4a^, p63 and Bmi-1 protein levels in PTKs and NHKs grown in KGM. As compared with NHKs, immunoblot showed an earlier induction of p16^INK4a^ in PTKs and subsequently a decrease of p63 and Bmi-1 protein levels (Fig. 3A). In keeping with these findings, the cytochemical investigation of the senescence-associated (SA)-ß-galactosidase activity revealed that control keratinocytes at P3 show a low percentage (7.4%) of positive cells, whilst this percentage raises to 36% in PTK-2 at the same passage (Fig. 3B).

Autophagy is an intricate catabolic process to clear unnecessary or dysfunctional cellular components in response to a variety of exogenous and endogenous stressors, including senescence triggers (37–39). In addition, multiple lines of evidence pointed out the dual function of autophagy as both activator and inhibitor of senescence mechanisms, in a cell- and context-dependent manner (39,40). In senescent keratinocytes, autophagy intensity determines two opposite outcomes: the majority of cells show high autophagy levels which commit them to programmed cell death driven by reactive oxygen species (41,42), whilst a minimal percentage of senescent cells, typified by reduced autophagic activity, can re-enter into the mitotic cycle (43). To explore the possible relationship between replicative senescence and autophagy in primary EBS-KLHL24 keratinocytes, we assessed the autophagic flux in PTKs and NHKs. To this aim, we measured the levels of autophagy markers p62, LC3B and GABARAP in basal conditions and after treatment with bafilomycin A1 (BafA1), an inhibitor of the autophagosome-lysosome fusion (44). While p62 accumulation upon BafA1 treatment was enhanced in one out of two patients, GABARAP and LC3B levels did not show significant differences in autophagy levels between patient and control cells (Supplementary Material, Fig. S2B).

### KLHL24 degrades a set of keratins expressed in primary foetal keratinocytes through a proteasome-dependent mechanism

**Figure 4:**
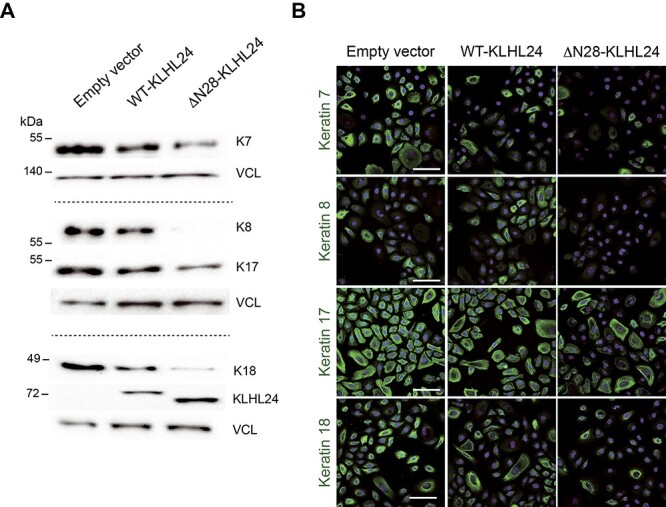
Foetal keratin protein levels are strongly reduced in primary foetal keratinocytes transduced with the mutant KLHL24 form. (**A**) Immunoblot (IB) showing keratin 7 (K7) (upper panel), K8, K17 (middle panel) and K18 (lower panel) protein levels in primary foetal keratinocytes (NHK-Fet) transduced with either control lentiviral (LV) particles (empty vector) or with LV particles expressing the wild-type (WT-KLHL24) or the truncated (ΔN28-KLHL24) forms of the KLHL24 protein. Representative blots of two independent LV transduction experiments showing similar results. Square brackets include distinct IB experiments. Note that KLHL24 protein levels are increased in keratinocytes expressing the ΔN28-KLHL24 as compared with the wild-type protein. Vinculin (VCL) was used as loading control. (**B**) Confocal microscopy analysis of K7, K8, K17 and K18 in NHK-Fet transduced with empty vector, WT-KLHL24 and ΔN28-KLHL24. Hoechst blue staining was used for nuclear labelling. Scale bar = 80 μm.

As EBS-KLHL24 patients are borne with congenital diffuse skin defects and their skin fragility rapidly ameliorates after birth, we hypothesized that one or more keratins expressed by keratinocytes during foetal epidermal development could represent additional KLHL24 targets. We selected keratin 7 (K7), K8, K17, K18 and K19 as candidates based on their known expression in foetal keratinocytes both *in vivo* and *in vitro* (28–31). Commercially available primary keratinocytes isolated from foetal human skin (NHK-Fet) were transduced with lentiviral (LV) particles carrying cDNA expressing either the wild-type KLHL24 protein (WT-KLHL24) or its truncated counterpart (ΔN28-KLHL24). Empty LV particles were used as control of transduction. Transduced NHK-Fet were selected by puromycin treatment. Cell transduction was then confirmed by immunofluorescence (Supplementary Material, Fig. S3A) and immunoblotting (IB) (Fig. 4A) assessment of wild-type and mutant KLHL24 protein.

Evaluation of candidate keratins by IB showed that K7, K8, K17 and K18 are all markedly down-regulated in NHK-Fet transduced with WT-KLHL24 or ΔN28-KLHL24 as compared to cells transduced with the empty vector (Fig. 4A), while K19 protein levels were not reduced (not shown). As expected, the ΔN28-KLHL24 protein produced stronger effects as compared with WT-KLHL24, indicating that its increased stability entails a greater ability to degrade its targets (15,16). In accordance with IB results, confocal microscopy analysis confirmed a reduction of K7, K8, K17 and K18 staining in WT- and ΔN28-KLHL24-transduced NHK-Fet, and a major decrease of foetal keratin staining in keratinocytes expressing the mutant KLHL24 form (Fig. 4B). On the other hand, K14 protein abundance did not appear different between mutant NHK-Fet and controls (Supplementary Material, Fig. S4). However, an altered spatial organization of K14 was detected in foetal keratinocytes expressing ΔN28-KLHL24 (Supplementary Material, Fig. S4), in line with our confocal microscopy findings in PTKs (Fig. 1B).

**Figure 5:**
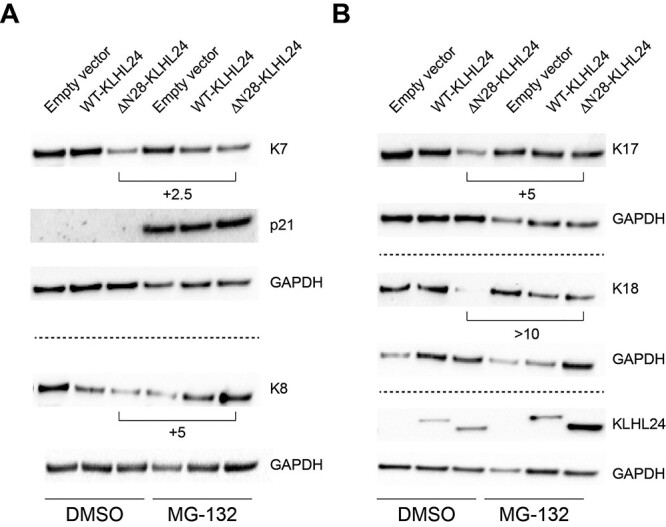
Proteasome inhibition rescues foetal keratins from the degradation mediated by the mutant KLHL24. (**A**) Immunoblot (IB) showing protein levels of keratin 7 (K7) and K8 in foetal keratinocytes (NHK-Fet) transduced with either control lentiviral (LV) particles (empty vector) or LV particles expressing either the wild-type (WT-KLHL24) or the truncated (ΔN28-KLHL24) forms of the KLHL24 protein. Transduced cells were treated with the proteasome inhibitor MG-132. The vehicle dimethyl sulfoxide (DMSO) was used as control. (**B**) IB showing protein levels of K17 and K18 in NHK-Fet transduced with either control LV particles (empty vector) or LV particles expressing WT-KLHL24 or ΔN28-KLHL24. KLHL24 was used as indicator of effective LV transduction. p21 accumulation in MG-132-treated cells was used as indicator of effective proteasome inhibition (86). Fold change variations between ΔN28-KLHL24-transduced cells in the presence or absence of MG-132 were calculated using Image Lab Software (Bio-Rad). Dotted lines separate distinct IB experiments. Representative blots of two independent LV transduction experiments showing similar results. Glyceraldheyde-3-phosphate dehydrogenase (GAPDH) was used as loading control.

Finally, in order to understand whether the KLHL24-dependent degradation of K7, K8, K17 and K18 is mediated by the ubiquitin-proteasome system, we treated the transduced NHK-Fet with the proteasome inhibitor, MG-132. K7, K8, K17 and K18 resulted markedly increased in NHK-Fet transduced with ΔN28-KLHL24 and treated with MG-132 as compared with cells expressing the mutant KLHL24 form and treated with the vehicle dimethyl sulfoxide (Fig. 5). In addition, we observed that both WT- and ΔN28-KLHL24 protein levels are augmented in MG-132-treated cells, confirming the presence of additional active ubiquitination sites beyond those present in the first 28 amino acids at the N-terminal end (16).

Taken together these findings indicate that a set of keratins expressed in NHK-Fet including K7, K8, K17 and K18 are subjected to a KLHL24-mediated degradation via the ubiquitin-proteasome system.

### Primary foetal keratinocytes expressing the mutant KLHL24 protein are more vulnerable to the heat-induced damage of the keratin network

In keratinocytes, heat stress disrupts the physiological keratin network, leading to a powerful, though rapidly reversible, keratin disassembly, fragmentation and perinuclear collapse (45–47). Furthermore, heat-stressed keratinocyte cell lines from EBS patients carrying *KRT5* and *KRT14* mutations exhibit a cytoskeletal damage featured by keratin aggregates distributed perinuclearly (46,48,49). Of note, aberrations in the keratin network are more evident in cell lines from EBS patients bearing the most detrimental *KRT5* mutations (48). In line with these findings, immortalized keratinocytes from EBS-KLHL24 patients showed cytoskeletal keratin alterations, which were more pronounced in heat stress conditions with respect to cells from healthy individuals (15).

**Figure 6:**
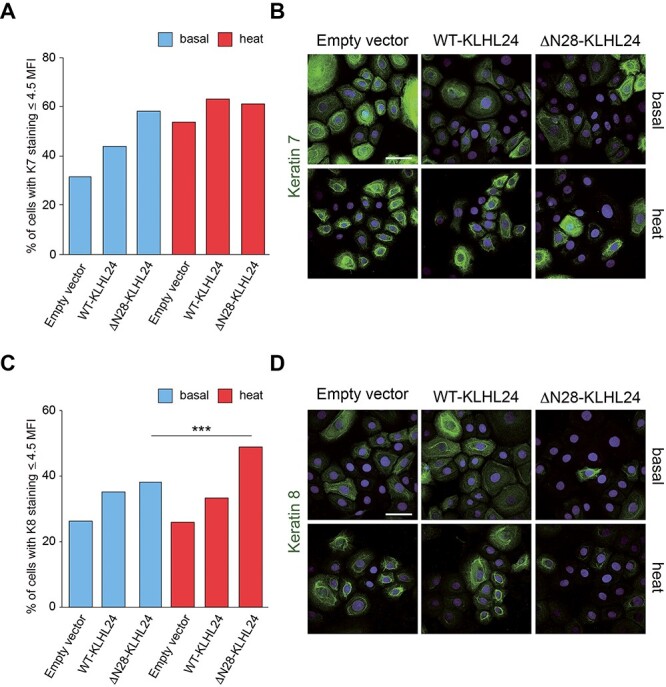
Keratin 7 and keratin 8 staining patterns in foetal keratinocytes transduced with wild-type and mutant KLHL24 protein and subjected to heat stress. (**A**, **C**) Histograms showing the percentage of foetal keratinocytes (NHK-Fet) displaying absent or barely detectable K7 (panel a) and K8 (panel c) staining (Mean Fluorescence Intensity ≤ 4.5, see Materials and Methods for details). K7 and K8 staining was investigated by confocal microscopy in NHK-Fet transduced with either control lentiviral (LV) particles (empty vector) or with LV particles expressing either the wild-type (WT-KLHL24) or the truncated (ΔN28-KLHL24) KLHL24 protein, in the presence or absence of heat stress. ‘Basal’ refers to control cells maintained at 37°C, ‘heat’ indicates heat-stressed cells. Statistical significance was calculated between ΔN28-KLHL24 subjected to heat stress and ΔN28-KLHL24 maintained in basal conditions. For each experimental condition, values are expressed as the ratio between the number of NHK-Fet showing MFI ≤ 4.5 for a given keratin and the number of cells counted in at least four random confocal images acquired with 40X objective. ^*^^*^^*^P < 0.001. Single-cell MFI was calculated by ImageJ Software (NIH). (**B**, **D**) Representative confocal microscopy images showing K7 (panel b) and K8 (panel d) staining in each experimental condition. Hoechst dye was used for nuclear staining. All images are maximum intensity projection of z-series. Scale bar = 50 μm. Note that in the presence of hyperthermic stress, NHK-Fet showed a greater proportion of cells displaying an altered keratin pattern as compared to their untreated counterpart, mainly visualized as a tendency to aggregate around the nucleus and an inhomogeneous cytoplasmic labelling, suggestive of heat-induced keratin clumping.

**Figure 7:**
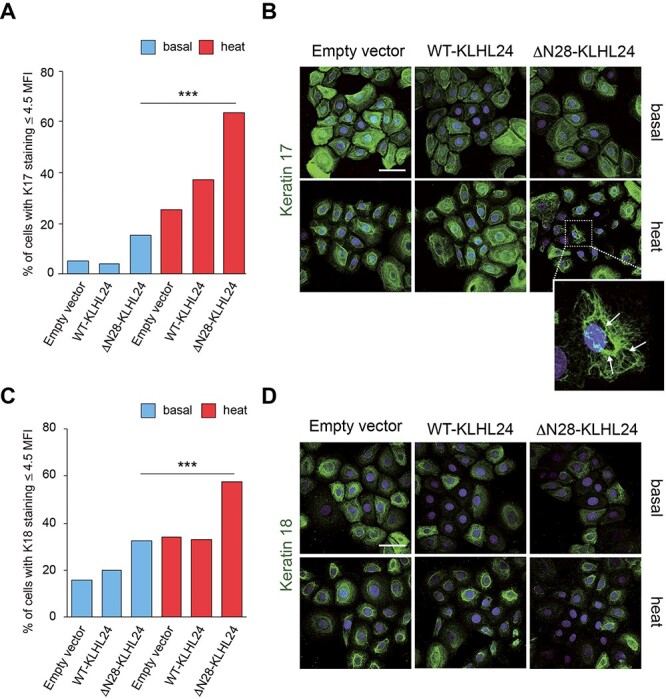
Keratin 17 and keratin 18 staining intensity and patterns in foetal keratinocytes transduced with wild-type and mutant KLHL24 protein and subjected to heat stress. (**A**, **C**) Histograms showing the percentage of foetal keratinocytes (NHK-Fet) displaying absent or barely detectable K17 (panel a) and K18 (panel c) staining (Mean Fluorescence Intensity ≤ 4.5, see Materials and Methods for detail). K17 and K18 staining was investigated by confocal microscopy in NHK-Fet transduced with either control lentiviral (LV) particles (empty vector) or LV particles expressing either the wild-type (WT-KLHL24) or the truncated (ΔN28-KLHL24) KLHL24 protein, in the presence or absence of heat stress. ‘Basal’ refers to control cells maintained at 37°C, ‘heat’ indicates heat-stressed cells. Statistical significance was calculated between ΔN28-KLHL24 subjected to heat stress and ΔN28-KLHL24 maintained in basal conditions. For each experimental condition, values are expressed as the ratio between the number of NHK-Fet showing MFI ≤ 4.5 for a given keratin and the number of cells counted in at least four random confocal images acquired with 40X objective. ^*^^*^^*^P < 0.001. Single-cell MFI was calculated by ImageJ Software (NIH). (**B**, **D**) Representative confocal microscopy images showing K17 (panel b) and K18 (panel d) staining in each experimental condition. Hoechst dye was used for nuclear staining. All images are maximum intensity projection of z-series. Scale bar = 50 μm. Note that in the presence of hyperthermic stress, NHK-Fet showed a greater proportion of cells displaying an altered K17 staining pattern as compared to their untreated counterpart, mainly visualized as a tendency to aggregate around the nucleus and an inhomogeneous cytoplasmic labelling (Fig. 7B, inset).

With the aim to evaluate the impact of the ΔN28-KLHL24 also in stress conditions, we assessed the intensity and distribution of K7, K8, K17 and K18 staining by confocal microscopy in transduced NHK-Fet subjected to heat shock by immersion for 15 min in a water bath set at 43°C. Single-cell mean fluorescence intensity (MFI) was calculated for each foetal keratin assayed in transduced NHK-Fet in the presence or absence of heat stress. In detail, a MFI value of 4.5 was used as threshold parameter to identify cells with an absent or barely detectable keratin staining. Notably, ΔN28-KLHL24-transduced cells subjected to heat stress showed a significant increase in the percentage of cells displaying absent to very low K8, K17 and K18 labelling (MFI ≤ 4.5) as compared with ΔN28-KLHL24-transduced cells maintained at 37°C (basal condition) (Figs 6 and 7). As for K7, the percentage of cells with very faint keratin staining did not increase in heat-treated ΔN28-KLHL24, likely due to the strikingly reduced K7 staining already in basal conditions (*i.e.* 54% of ΔN28-KLHL24-transduced NHK-Fet showed absent or barely detectable K7 staining) (Fig. 6A). K17 appeared as the most susceptible keratin to heat-mediated damage already after transduction with the empty vector. Nevertheless, the concomitant presence of heat stress and mutant KLHL24 protein induced a marked increase (+48%) in the percentage of NHK-Fet hallmarked by scant K17 staining as compared with the counterpart maintained at 37°C. Furthermore, ΔN28-KLHL24-transduced NHK-Fet challenged with hyperthermic stress displayed a disorganized K17 network consisting of large negative staining cytoplasmic areas surrounded by condensed keratin, whilst in the remaining conditions K17 labelling appeared more uniformly distributed (Fig. 7B, inset).

**Figure 8:**
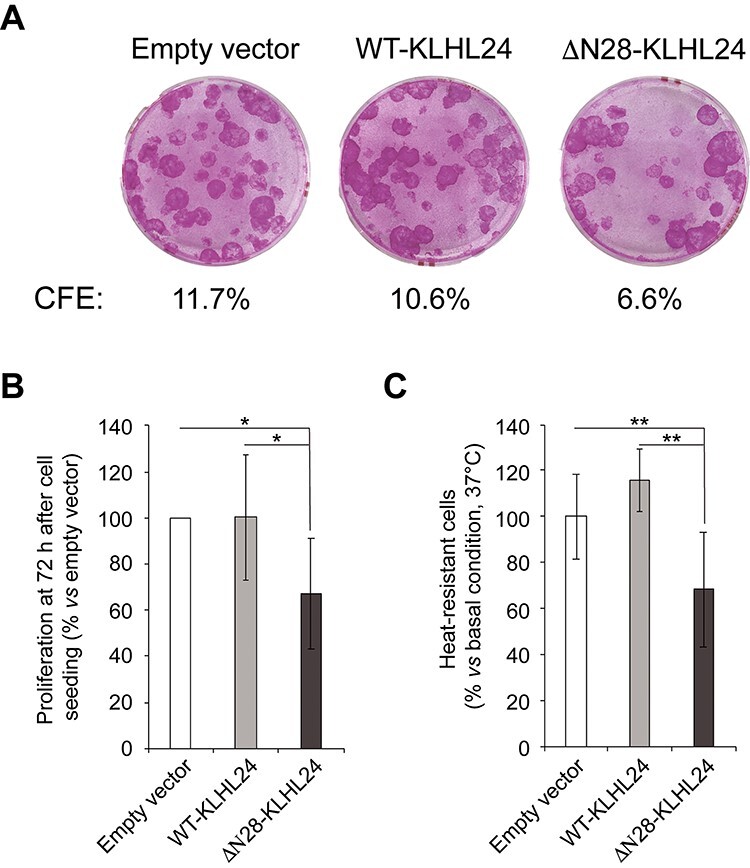
Mutant KLHL24 impairs foetal keratinocyte colony forming efficiency, proliferation and resilience to heat stress. (**A**) Representative images showing colony forming efficiency (CFE) of foetal keratinocytes transduced with control lentiviral particles (empty vector), wild-type KLHL24 (WT-KLHL24) and mutant KLHL24 (ΔN28-KLHL24). CFE was performed at the first passage after lentiviral infection. The percentage of CFE is indicated for each experimental condition, and expressed as the ratio of the number of colonies on the number of seeded keratinocytes (500 cells/dish). (**B**) Histogram showing the proliferative rate of foetal keratinocytes expressing WT-KLHL24 or ΔN28-KLHL24 with respect to cells transduced with the control lentiviral particles (empty vector). To measure proliferative ability, the MTT assay was performed after 72 h from cell seeding. Absorbance (Abs) values were converted in percentage by using the following formula: Abs_WT- or ΔN28-KLHL24_ x 100/Abs_empty vector._ Data are presented as mean ± SD of at least six technical replicates per sample. ^*^P < 0.05. (**C**) Histogram showing the ability of foetal keratinocytes transduced with WT-KLHL24 or ΔN28-KLHL24 to overcome heat stress with respect to control cells (empty vector). Plates containing subconfluent keratinocytes were immersed for 15 min in a water bath set at 43°C, washed two times with PBS and stained with a crystal violet (CV) solution. Thereafter cells were lysed to release the dye and absorbance values, suggestive of the number of adherent cells, were quantified by Benchmark Plus Microplate Spectrophometer System (Bio-Rad). Data indicates the percentage of adherent cells ± SD of at least six technical replicates per sample with respect to cells maintained at 37°C (basal conditions). Absorbance (Abs) values were converted in percentage by using the following formula: Abs _43°C_ × 100/Abs _37°C_. ^*^^*^P < 0.01.

### Primary foetal keratinocytes expressing the mutant KLHL24 protein display reduced colony forming efficiency, proliferative rate and diminished resilience to heat-stress

To investigate whether the mutant KLHL24 protein impairs keratinocyte functions, we assessed colony forming efficiency, proliferation rate and resilience to heat-stress of NHK-Fet transduced with LV particles expressing the wild-type or the mutant KLHL24 protein. In line with our findings in PTKs (Fig. 2A and B), CFE assay revealed a reduced clonogenicity in ΔN28-KLHL24-transduced NHK-Fet with respect to control cells (Fig. 8A). Specifically, mutant cells showed an average CFE decrease of 40% as compared to empty vector- and wild-type KLHL24-transduced cells (Fig. 8A).

After 72 h from cell seeding, MTT viability assay revealed that NHK-Fet expressing the ΔN28-KLHL24 protein present lower absorbance values, suggestive of a diminished proliferative ability, as compared with control cells treated with empty LV particles (empty vector) and cells expressing the wild-type protein. Specifically, ΔN28-KLHL24-transduced cells exhibited a statistically significant 33% reduction of cell viability with respect to control cells and WT-KLHL24-transduced cell (Fig. 8B).

To evaluate the impact of mutant KLHL24 protein on the cell resilience to thermic stress, subconfluent transduced NHK-Fet were subjected to heat-shock as previously described. After two washing steps with PBS, adherent cells were quantified by crystal violet (CV) staining. A significant reduction in the number of adherent cells was observed in heat-treated ΔN28-KLHL24-transduced cells as compared with their counterpart maintained in basal conditions (37°C). In detail, cells containing the empty vector or expressing the WT-KLHL24 didn’t suffer the hyperthermia-induced detachment, whilst about the 30% of keratinocytes transduced with the ΔN28-KLHL24 protein detached from the plate after heat-stress (Fig. 8C).

Taken together, these analyses indicate that the mutant KLHL24 protein affects foetal keratinocyte proliferation and impairs cell ability to overcome heat stress.

## Discussion

EBS-KLHL24 is determined by dominant gain-of-function mutations in the translation initiation codon of the gene encoding for the cullin 3 (CUL3)-RING E3 ligase KLHL24. The mutant protein (ΔN28-KLHL24) is more refractory to the self-catalysed ubiquitin-mediated turnover, and thus more efficient in degrading its substrates (15,16,24). EBS-KLHL24 newborns are hallmarked by severe aplasia cutis congenita (ACC) and skin fragility, which arise during the foetal development. ACC can occur in all EB types, it usually involves lower extremities and is considered secondary to *in utero* friction (50). Remarkably, EBS-KLHL24 is the only EB subtype in which ACC has been observed in all patients described to date, regularly affecting both lower and upper extremities and sometimes also the trunk (15,16,21–24). Of note, skin fragility of EBS-KLHK24 rapidly improves, frequently already in infancy. However, healing of congenital skin defects in EBS-KLHL24 patients results in skin atrophy with a typical stellate scarring pattern on a background of hypo- or hyperpigmented macules: these features are reminiscent of aging-related stellate pseudoscars. In addition, follicular atrophoderma with hair loss frequently occurs at skin atrophy sites, accompanied in some patients by development over time of alopecia affecting terminal hair. Among EB types, this constellation of clinical features is unique to EBS-KLHL24 (15,16,21,22,50,51). Of note, cardiac anomalies are present in the vast majority of EBS-KLHL24 individuals, who develop an early-onset dilated cardiomyopathy that can evolve fatally if not timely diagnosed and managed (23,24). On the other hand, loss-of-function mutations lying within two different KLHL24 functional domains have been recently shown to cause a recessively inherited form of hypertrophic cardiomyopathy, characterized by polyglucan, glycogen and desmin accumulation (17).

### Foetal keratins are heat-sensitive substrates of the ubiquitin-ligase KLHL24

Phenotypic and pathological findings in EBS-KLHL24 and KLHL24-dependent hypertrophic cardiomyopathy resulted in the identification of intermediate filaments (IFs) as candidate substrates of KLHL24-mediated degradation in the skin and heart, respectively. Specifically, the basal keratin 14 (K14) represents the main KLHL24 target in keratinocytes, whilst desmin—an IF expressed in muscle cells—has been recently identified as KLHL24 substrate in human induced pluripotent stem cells (hiPSC)-derived cardiomyocytes (15–17,27). However, K14 expression is ubiquitous in basal keratinocytes during foetal development and postnatal life. Thus, its enhanced degradation by KLHL24 would not explain the striking congenital skin manifestations and their rapid amelioration after birth in EBS-KLHL24 patients (52). In addition, the present study and previous reports agree in describing unaltered K14 protein levels in EBS-KLHL24 patient biopsies and primary keratinocytes, though mutant cells display a morphologically altered K14 network (15), which we also detected in human foetal keratinocytes expressing ΔN28-KLHL24. Similarly to K14 in keratinocytes, the vimentin network is altered in primary fibroblasts from EBS-KLHL24 patients but its protein levels are comparable to control cells (15). Altogether these results point to additional KLHL24 substrates contributing to skin fragility in foetal life. Our findings demonstrate that K7, K8, K17 and K18—a set of keratins expressed during foetal skin development (28–31)—are strongly degraded *via* proteasome in primary human foetal keratinocytes expressing ΔN28-KLHL24 as compared to cells transduced with the wild-type form or control empty lentiviral particles. The outlined degradation by KLHL24 of a wide set of keratins, ranging from those typically expressed during foetal skin development to K14 (15,16), is supported by the emerging role of KLHL24 as a pan-regulator of IFs. This feature is shared with other Kelch family members (*i.e.* KLHL9 and KLHL16), which are directly or indirectly involved in cytoskeletal IFs turnover in a variety of cell types (9,10,53–57). Furthermore, confocal microscopy analysis revealed that the keratin network of ΔN28-KLHL24-transduced cells appears more sensitive to heat-induced damage, with a large percentage of cells displaying absent or barely detectable K8, K17 and K18 staining in response to hyperthermia. In addition, we observed that ΔN28-KLHL24 impairs NHK-Fet proliferation rate and resilience to heat-stress. In the latter case, heat treatment resulted in a significant reduction in the number of adherent ΔN28-KLHL24-tranduced cells as compared to the remaining experimental conditions. In our hypothesis, the mutant KLHL24 protein leads to a general keratin network weakening, which results in a reduced NHK-Fet proliferation (58,59) and increased cell vulnerability to heat stress (46,48,49). However, we cannot exclude a direct, detrimental role of KLHL24 on cell proliferation and adhesion in stress conditions.

During all the intrauterine life, foetal keratinocytes are subjected to peculiar chemical–physical conditions including their continuous exposure to amniotic fluid—a complex and dynamic milieu with emerging roles in foetal skin formation and function (60–62). Interestingly, the environmental stimuli challenging keratinocytes during foetal development are recognized to play a pathogenetic role in other congenital skin diseases, such as self-healing collodion baby, characterized by a dramatic amelioration of phenotype shortly after birth (63–65). As foetal body temperature constantly exceeds the maternal one by 0.3–0.5°C (66), our laboratory findings on mutant keratinocytes subjected to heat stress may suggest a relationship between the in utero conditions (*i.e.* foetal temperature, amniotic fluid hydrostatic pressure and composition) and the severe congenital skin defects observed in EBS-KLHL24 patients. However, future studies are needed to confirm the contribution of the intrauterine environment to such a peculiar disease phenotype.

### Premature replicative senescence in primary keratinocytes from EBS-KLHL24 as a clue to skin atrophy in patients

Cutaneous atrophy, scarring and pigmentary abnormalities are defects observed in EBS-KLHL24 individuals (15,16,21,22,24). However, the molecular bases of these distinctive phenotypic traits as well as the involvement of mutant KLHL24 protein in keratinocyte aging are not fully elucidated. He and coll. Tried to explain skin atrophy and scarring in EBS-KLHL24 patients by the augmented phosphorylation of the kinase p38 and a two-fold increased apoptosis observed in immortalized keratinocytes from patients after heat stress and, to a lesser extent, also in basal conditions (15). A different line of research relying on the KLHL24 induction by the pro-differentiation transcription factor p73 (16,67), suggests a direct involvement of KLHL24 in keratinocyte differentiation (16,68). Nevertheless, the putative relationship between an enhanced EBS-KLHL24-dependent keratinocyte differentiation process and skin senescence in patients remains to be investigated (33).

In the present study, we showed the premature activation of replicative senescence in EBS-KLHL24 primary keratinocytes grown on a 3T3-J2 fibroblast feeder-layer, *i.e.* in culture conditions that faithfully reproduce those of human epidermis *in vivo* (69). In detail, lifespan and CFE were significantly reduced in PTKs as compared to NHKs, with divergences between patient and control CFE already evident in cells isolated from biopsy (P0). In addition, the percentage of paraclones rapidly increased in serial subcultures of PTKs with respect to NHKs. In accordance with these findings, we observed an early induction of p16^INK4a^, a well-established marker of keratinocyte aging and stem cell depletion, and a reduction of the stemness markers Bmi-1 and p63 in PTKs as compared with NHKs (33). Finally, PTKs grown in defined medium were positive to the SA-β-galactosidase assay, and showed an enlarged, flattened and vacuolized cell morphology, all typical traits of senescent, terminally differentiated cells (70,71). Senescent features of EBS-KLHL24 keratinocyte well correlate with the peculiar skin atrophy observed in patients *in vivo*.

Our findings revealed that ΔN28-KLHL24-transduced NHK-Fet and PTKs share defects in clonogenicity and proliferation rate, suggesting that PTK aberrations could arise from a direct role of KLHL24 in reducing clonogenicity, or as a consequence of the enhanced degradation of keratin network (59,74). However, similarly to what described for other EB types (72,73), we cannot exclude that the diminished clonogenic potential of PTKs could be fostered also by an accelerated depletion of keratinocyte stem cells, resulting from the severe skin fragility and recurrent lesions challenging foetal keratinocytes in the prenatal life. Thus, the molecular determinants underlying the early replicative senescence and accelerated clonal conversion in PTKs remain to be characterized in detail. Finally, premature senescence in PTKs could play a role also in patient skin dyspigmentation features, through keratinocyte-melanocyte cross-talk (75,76).

In conclusion our findings identify foetal keratins as novel KLHL24 targets which could underlie congenital skin defects and also indirectly contribute to the injury-driven postnatal skin atrophy in EBS-KLHL24. Future studies will allow to determine if foetal keratin degradation by KLHL24 occurs via direct or indirect mechanisms and to identify possible additional KLHL24 substrates in different tissues, such as the nervous system, putatively involved in the pathogenesis of the syndromic phenotype of EBS-KLHL24 patients.

## Materials and Methods

### Patient samples, immunofluorescence and molecular genetic analyses

Two children affected with EBS-KLHL24 were studied (PT-1 and PT-2). PT-1 has been described (Case 1, (22)), while PT-2 is a previously unreported 7-year-old female child, born to healthy non consanguineous healthy parents. Specifically, EBS-KLHL24 biopsies were taken from perilesional unblistered skin following skin rubbing, which results in basal keratinocyte vacuolization and microblistering, only detectable at microscopy analysis. Patient skin biopsies and blood samples were obtained after informed consent, with the approval of the Ethics Committees of participating Institutions and in conformity with the Helsinki guidelines. Normal human keratinocytes (NHKs) were obtained from skin or foreskin of age-matched healthy subjects undergoing surgery.

Skin biopsies were used for immunofluorescence antigen mapping and keratinocyte cultures as described (77). PT-2 genomic DNA was extracted from peripheral blood using QIAsymphony DSP DNA Mini Kit (Qiagen, Hilden, Germany), and sequence variants were identified through Next Generation Sequencing (NGS) approach (NimbleGenSeqCap Target Enrichment—Roche, Madison, WI, USA; Twist Human Core Exome Kit—Twist Bioscience, San Francisco, CA, USA) and NextSeq550 sequencing platform (Illumina, San Diego, CA, USA). Variant validation and segregation analysis were performed by Sanger sequencing.

### Cell cultures and treatments

PTKs and NHKs were cultivated on a feeder layer of lethally irradiated 3T3-J2 murine fibroblasts (Kerafast, Boston, MA, USA) in DMEM and Ham’s F12 media (3:1 mixture) containing 10% of foetal calf serum and supplements as described (78). In selected experiments, keratinocytes were cultured in defined serum-free, calcium-free Keratinocyte Growth Medium (KGM—Lonza, Basel, Switzerland). Defined serum-free, calcium-free growth media have been developed for the selective growth of keratinocytes in the absence of feeder layer, maintaining them in a proliferative state (79,80). NHK-Fet were purchased from ScienCell Research Laboratories (Carlsbad, CA, USA) and cultured in Keratinocyte Fetal Medium (ScienCell) as described by the manufacturer. All cells were used at passages 1–4. The following compounds were used: MG-132 at 1 μM for 24 h (Merck, Darmstadt, Germany) and bafilomycin A1 (BafA1) at 20 nM for 24 h (Merck).

### Colony forming efficiency (CFE) and lifespan

For CFE assay, PTKs (PTK-1 and -2), NHKs (NHK-1 and -2) and transduced NHK-Fet were plated on 3T3-J2 fibroblasts, colonies were fixed 14 days later with rhodamine-B (Merck) and counted as described (32,81). Total colonies were calculated as percentage of total plated cells (number of colonies x 100/number of cells plated) and paraclones as the ratio of aborted colonies to the total number of colonies (32,81). For lifespan, PTKs and NHKs were passaged until senescence and the number of cell generations was calculated using the following formula: x = 3.322 log N/No., where N is the total number of cells obtained at each passage and No. is the number of clonogenic cells. Clonogenic cells were calculated from CFE data, which were determined separately in parallel dishes at the time of each cell passage. The cumulative number of cell generations per passage was plotted against total time in culture (33).

### Senescence associated β-galactosidase assay

PTKs and NHKs were seeded onto 24-well plates (5 x 10^4^ cells per well) and cultured in KGM until subconfluence. Thereafter, keratinocytes were fixed and stained with the senescence associated-β-galactosidase (SA-β-gal) assay kit (Cell Signaling Technology, Danvers, MA, USA) following manufacturer’s instructions. Plates were incubated in a dry incubator at 37°C for three days. The development of blue colour was checked by using a Leica DMi8 inverted microscope (Leica, Wetzlar, Germany). The number of SA-β-gal-positive cells was evaluated by counting 10 random fields at 400X magnification and represented as a percentage of total cell number.

### Expression plasmid generation

RNA purified from NHK-1 (P0) was used as transcription template to obtain the 1875 basepair (bp) long wild-type KLHL24 form (WT-KLHL24) and its mutant counterpart (ΔN28-KLHL24). In the latter case, we amplified a KLHL24 sequence lacking the first 84 nucleotides of the coding region, which results into the truncated protein form. PCR amplification was performed using Q5 high-fidelity DNA polymerase (New England BioLabs, Ipswich, MA, USA) and primers indicated in Supplementary Material, Table S1. Specifically, all oligonucleotides were designed on the KLHL24 mRNA transcript variant 1 (NM_001349413.1). PCR products were purified and cloned between NheI and NotI restriction sites in pcDNA3.1-HA vector carrying a N-terminal Hemagglutinin (HA)-tag (82). Due to the reported low KLHL24 expression levels (16), HA-tag was used as a backup plan to ensure its detection. WT-KLHL24 and ΔN28-KLHL24 cDNAs were amplified by PCR from the pcDNA3.1-HA vector using two oligonucleotides containing the recognition sites for the restriction endonuclease XhoI (XhoI-HA-Fw) and SalI (SalI-KLHL24-Rev) (Supplementary Material, Table S1). PCR products were purified and subcloned into a lentiviral vector carrying puromycin resistance in XhoI site (pLVX-M-puro, Addgene, Watertown, MA, USA) (83). The correct orientation was checked by sequencing.

### Foetal keratinocyte transduction with lentiviral vectors

Lentiviral supernatants were produced using standard procedures (84). Briefly, NHK-Fet (1 x 10^6^ cells) were seeded in 100 mm Petri dishes in Keratinocyte Fetal Medium. The day after, cells were infected with either control lentiviral (LV) particles (empty vector) or with LV particles expressing either WT-KLHL24 or ΔN28-KLHL24. Infection was performed for 2 h in the presence of 6 μg/mL of polybrene (Applied Biological Materials Inc, Richmond, bc, Canada) and Viralplus transduction enhancer (Applied Biological Materials) in 2.5 mL final volume. Then, 7.5 mL of fresh medium was added to the cells for 24 h. Afterwards cells were selected with 1.5 μg/mL puromycin (Merck). After 48 h, NHK-Fet were detached and seeded for protein extraction, confocal microscopy and other downstream analyses. Transduction efficiency was evaluated by real-time RT-PCR using oligonucleotides listed in Supplementary Material, Table S1, IB and confocal microscopy. In line with previous reports, our findings confirmed that the ΔN28-KLHL24 protein form is more abundant with respect to its wild-type counterpart due to the increased stability to the proteasome-mediated degradation (Supplementary Material, Fig. S3).

### Immunoblotting

Keratinocytes were lysed in 6.5 M urea buffer (85) supplemented with a mixture of phosphatase (Cocktail 2 and 3, Merck) and protease inhibitors (Merck). Proteins (5–15 μg) were run under reducing conditions using 4–12% precast polyacrylamide gels (Thermo Fisher Scientific, Waltham, MA, USA) or 10% polyacrylamide handmade gels, depending on the probed proteins. Nitrocellulose membranes were incubated with antibodies listed in Supplementary Material, Table S2. Detection was performed using Amersham ECL Prime (GE Healthcare Life Sciences, Little Chalfont, UK). Images were acquired with ChemiDoc™ XRS+ System (Bio-Rad, Hercules, CA, USA). Band density was evaluated using Image Lab Software (Bio-Rad). Glyceraldheyde-3-phosphate dehydrogenase (GAPDH) and vinculin (VCL) were used as loading controls.

### Confocal microscopy

Keratinocytes were cultured in KGM on glass coverslips. Subconfluent cells were fixed in ice-cold methanol for 10 min, saturated with 5% bovine serum albumin in PBS and incubated overnight at 4°C with specific primary antibodies (Supplementary Material, Table S2). The day after, coverslips were washed with PBS and incubated 1 h at room temperature with anti-rabbit or anti-mouse secondary antibodies conjugated to Alexa Fluor dyes (Supplementary Material, Table S2), together with Hoechst 33342 (Thermo Fisher Scientific) for nuclear staining. Images were collected with an Olympus Fluoview FV1000 Confocal Laser Scanning Microscope (Olympus, Tokyo, Japan) equipped with FV10-ASW version 4.1a software (Olympus), using 40X/0.90 or 60X/1.42 N.A. (Numerical Aperture) objectives. Fluorochrome unmixing was performed by acquisition of automated-sequential collection of multi-channel images, in order to reduce spectral crosstalk between channels. Z-series images were obtained through the collection of serial, confocal sections at 0.5 μm intervals. To compare confocal data from different specimens, identical confocal settings were used for image acquisition.

### Analysis of staining patterns in transduced cells subjected to heat stress

Transduced foetal keratinocytes were seeded at a density of 3 x 10^4^ cells per well in 24-well plates containing glass coverslips. Keratinocytes were cultured in defined medium until sub-confluence, and then subjected to thermal stress as previously described with slight modifications (46). Briefly, growth medium was replaced at the start of heat-shock experiments with 43°C pre-warmed medium. Thereafter, culture plates were sealed and immediately immersed for 15 min in a water bath set at 43°C. Plates maintained at 37°C were used as control. At the end of the hyperthermic stress, adherent keratinocytes were washed with PBS and fixed with ice-cold methanol. Preparation of glass coverslips for the confocal microscopy analysis was performed as described above. Confocal images were collected by using a 40X objective and analysed. Single-cell Mean Fluorescence Intensity (MFI) was quantified using ImageJ software (NIH, Bethesda, MD, USA) for K7, K8, K17 and K18. MFI represents the sum of pixel intensities in the selected area, divided for the total number of pixels in the selection. A value of MFI ≤ 4.5 was selected as cut-off parameter to identify low-stained cells, *i.e.* cells showing absent or barely detectable keratin staining. The entire set of cells contained in a minimum of four random fields were collected by confocal microscopy by using a 40X objective and analysed.

### Functional assays on transduced cells

Proliferation rate of transduced foetal keratinocytes was assayed by MTT (3-(4,5-Dimethyl-2-thiazolyl)-2,5-diphenyl-2H-tetrazolium bromide). Briefly, 4 x 10^3^ NHK-Fet transduced with either control lentiviral (LV) particles (empty vector), or wild-type (WT-KLHL24) or mutant KLHL24 form (ΔN28-KLHL24) were seeded in 96-well plates and grown in defined medium. After 72 h, growth medium was replaced with MTT solution. The resulting formazan crystals were solubilized by dimethyl sulfoxide (DMSO) to release dye, and the colour intensity, which is proportional to cell number, was measured at 560 nm with an Infinite F50 microplate reader (Tecan, Mannedorf, Switzerland). Resilience to heat-stress in transduced NHK-Fet was assayed by a colorimetric approach. Briefly, transduced cells were growth in defined medium until subconfluence. Heat shock was performed as described above. At the end of the hyperthermic stress, adherent and heat-resistant keratinocytes were washed two times with PBS, stained with a 0.5% crystal violet (CV) solution and lysed with 1% sodium dodecyl sulfate (SDS) to release dye. The absorbance of the dye solution was measured by Benchmark Plus Microplate Spectrophometer System (Bio-Rad) at 595 nm. Transduced NHK-Fet maintained at 37°C (basal condition) were used as controls.

### Statistics

Unpaired Student’s t-test was used to calculate the statistical significance between two groups of data. Chi-square test was employed to evaluate the statistical significance of IF analyses on transduced keratinocytes subjected to thermal stress. P-values ≤ 0.05 were considered to be statistically significant.

*Conflict of Interest statement*: None.

## Supplementary Material

Supplementary_Fig_1_ddab318

Supplementary_Fig_2_ddab318

Supplementary_Fig_3_ddab318

Supplementary_Fig_4_ddab318

Supplementary_table_1_ddab318

Supplementary_table_2_ddab318
