# Supplementary figures and images for: Proteasome-mediated degradation of keratins 7, 8, 17 and 18 by mutant KLHL24 in a foetal keratinocyte model: Novel insight in congenital skin defects and fragility of epidermolysis bullosa simplex with cardiomyopathy
Source: Hum Mol Genet. 2021 Nov 5;31(8):1308–24. doi: 10.1093/hmg/ddab318 (PMC9029237; doi:10.1093/hmg/ddab318)

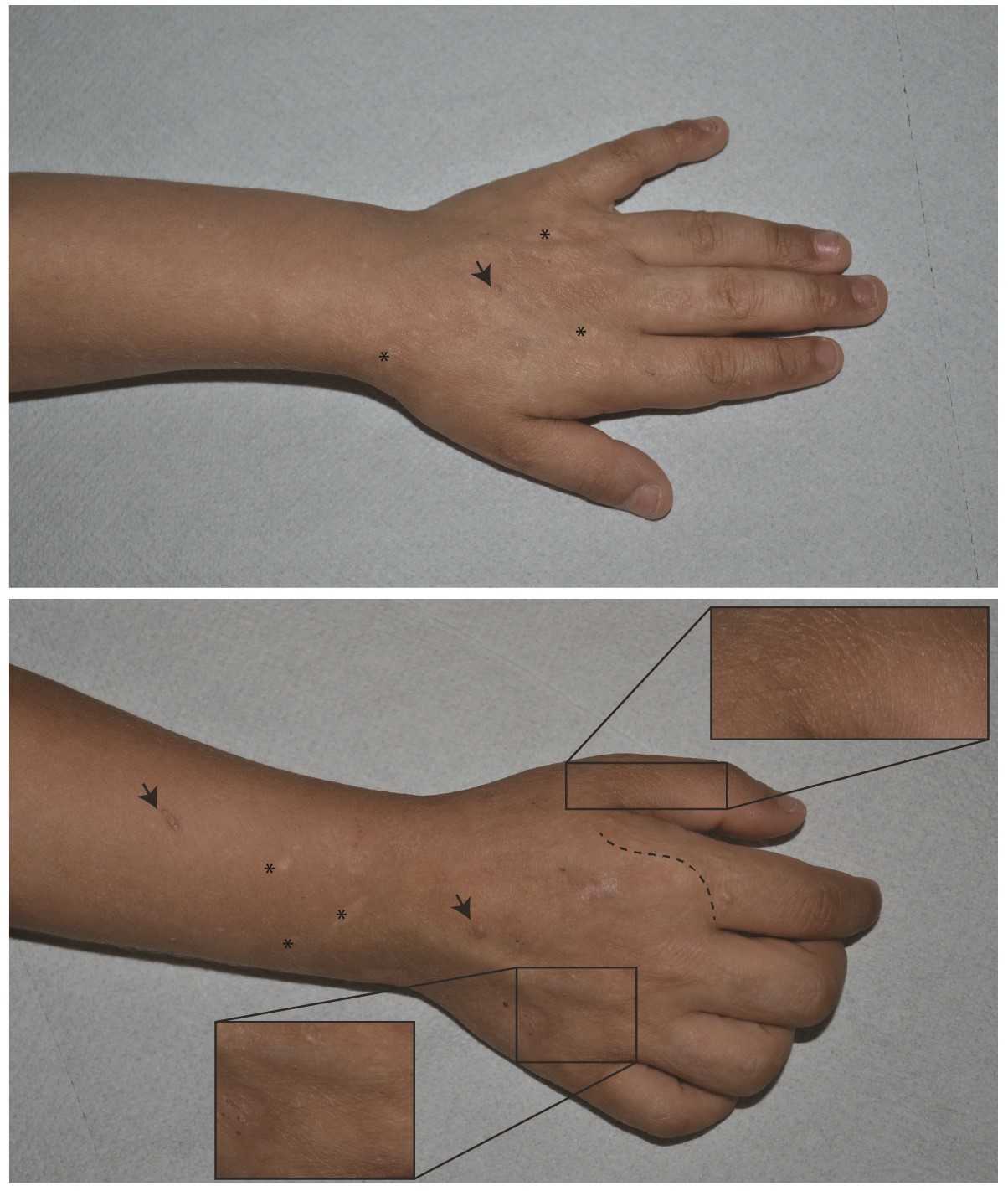

Supplement: Supplementary_Fig_1_ddab318 [file supplementary_fig_1_ddab318.jpeg]

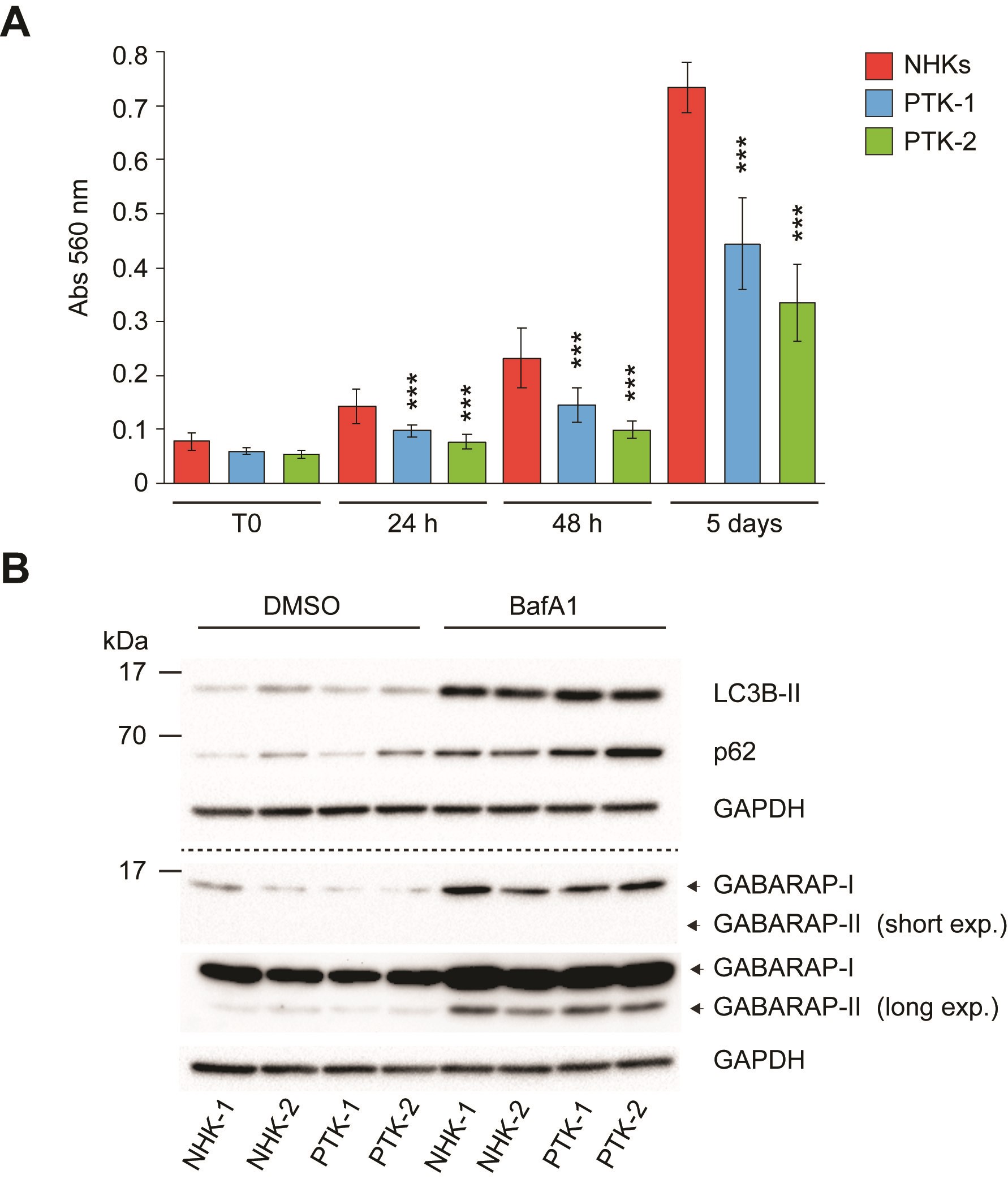

Supplement: Supplementary_Fig_2_ddab318 [file supplementary_fig_2_ddab318.jpeg]

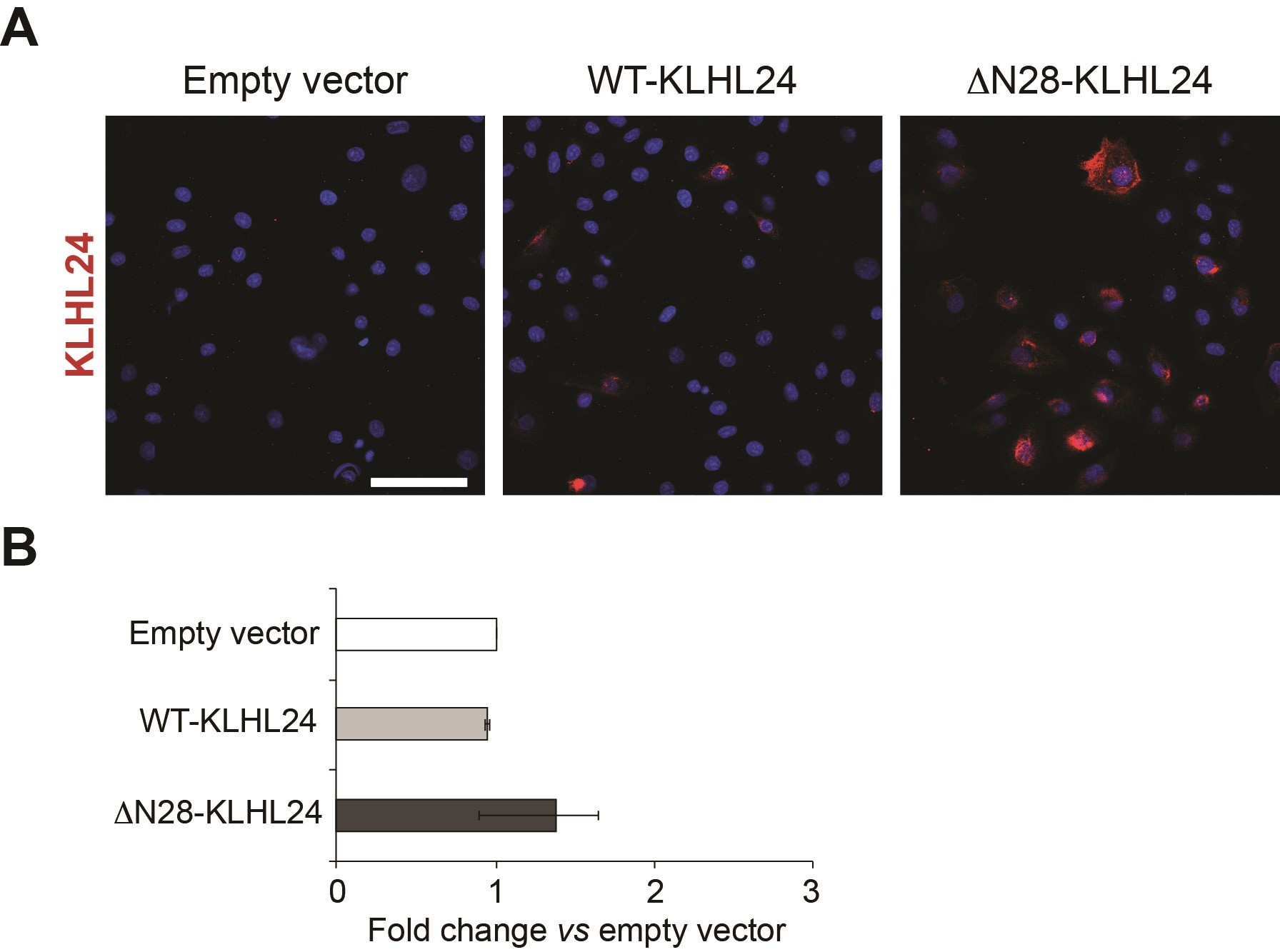

Supplement: Supplementary_Fig_3_ddab318 [file supplementary_fig_3_ddab318.jpeg]

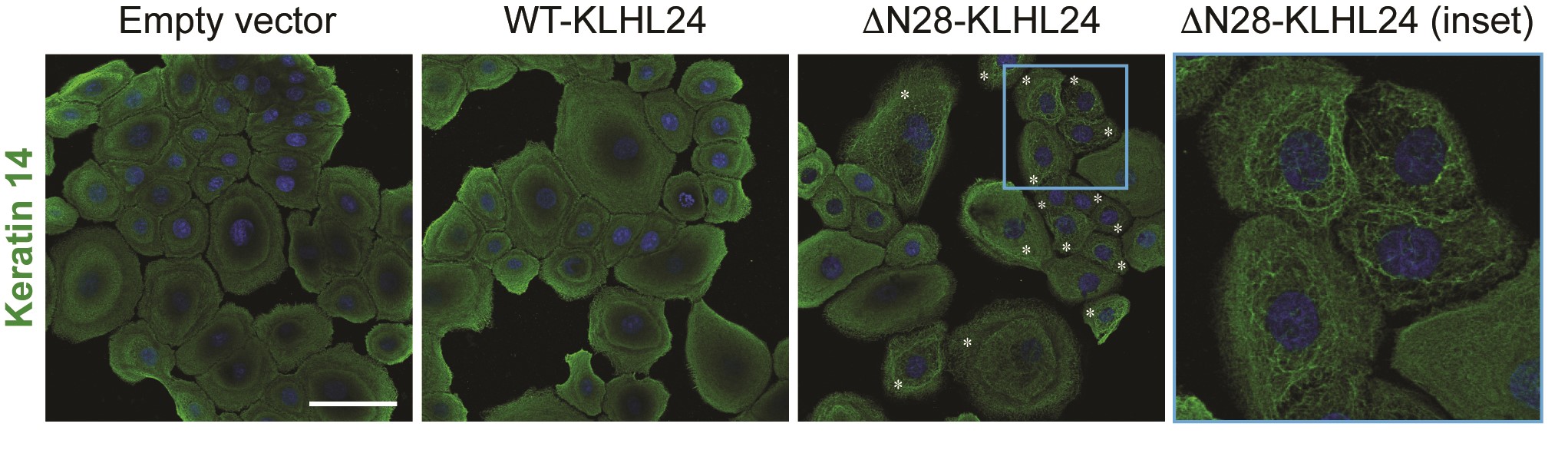

Supplement: Supplementary_Fig_4_ddab318 [file supplementary_fig_4_ddab318.jpeg]
