# Supplementary material for: Proteasome-mediated degradation of keratins 7, 8, 17 and 18 by mutant KLHL24 in a foetal keratinocyte model: Novel insight in congenital skin defects and fragility of epidermolysis bullosa simplex with cardiomyopathy
Source: Hum Mol Genet. 2021 Nov 5;31(8):1308–24. doi: 10.1093/hmg/ddab318 (PMC9029237; doi:10.1093/hmg/ddab318)
Supplement: Supplementary_table_1_ddab318 [file supplementary_table_1_ddab318.docx]

**Supplementary table 1**. **Primers used in this study.**

| **Primer name (cDNA)** | **Sequence (5’-3’)** |
| --- | --- |
| **NheI**-WT-KLHL24_fw | GGCC**GCTAGC**GTACTAATATTGGGACGCAGAC |
| **NheI**-ΔN28-KLHL24_fw | GGCC**GCTAGC**GACCCCAAATCTCTGACAGGTC |
| **NotI**-KLHL24_rev | GGCC**GCGGCCGC**TCAGAGTTTAAAGCATTTCTCATTG |
| **XhoI**-HA_fw | GGCC**CTCGAG**ATGGCTTACCCATACGATGTTCC |
| **SalI**-KLHL24_rev | GGCC**GTCGAC**AAGCGGCCGCTCAGAGTTTAAAGC |
| pLVX-M-puro_fw | GGCACCAAAATCAACGGGAC |
| pLVX-M-puro_rev | CCCACCGTACACGCCTACC |

Legend: Restriction sites are indicated in bold. Underlined text indicates a portion of the Hemagglutinin (HA)-tag nucleotide sequence. Abbreviations: fw, forward; rev, reverse; puro, puromycin.
