# Supplementary material for: Proteasome-mediated degradation of keratins 7, 8, 17 and 18 by mutant KLHL24 in a foetal keratinocyte model: Novel insight in congenital skin defects and fragility of epidermolysis bullosa simplex with cardiomyopathy
Source: Hum Mol Genet. 2021 Nov 5;31(8):1308–24. doi: 10.1093/hmg/ddab318 (PMC9029237; doi:10.1093/hmg/ddab318)
Supplement: Supplementary_table_2_ddab318 [file supplementary_table_2_ddab318.docx]

**Supplementary table 2. Antibodies used in this study.**

| **Primary Antibody**  Antigen | **Clone**  **(host species)** | **Company**  **and product code (cod.)** | **Application and dilution** |
| --- | --- | --- | --- |
| Bmi-1 | F6 (mouse) | Merck (cod. 05-637) | IB, 1:1000 |
| GABARAP | E1J4E (rabbit) | CST (cod. 13733) | IB, 1:1000 |
| GAPDH | 14C10 (rabbit) | CST (cod. 2118) | IB, 1:1000 |
| Keratin 5 | EP1601Y (rabbit) | Thermo Fisher Scientific  (cod. MA5-14473) | IB, 1:20000 |
| Keratin 7 | OV-TL12/30 (mouse) | Thermo Fisher Scientific  (cod. MA5-11986) | IB, 1:5000  IF, 1:500 |
| Keratin 8 | TS1 (mouse) | Thermo Fisher Scientific  (cod. MA5-14428) | IB, 1:5000  IF, 1:50 |
| Keratin 14 | LL002 (mouse) | Merck (cod. CBL197) | IB, 1:20000  IF, 1:500 |
| Keratin 17 | E3 (mouse) | Thermo Fisher Scientific  (cod. MA1-06325) | IB, 1:5000  IF, 1:100 |
| Keratin 18 | C-04 (mouse) | Abcam (cod. ab668) | IB, 1:5000  IF, 1:100 |
| KLHL24 | pAb (rabbit) | Thermo Fisher Scientific  (cod. PA5-63289) | IB, 1:100  IF, 1:50 |
| LC3B | D11 (rabbit) | CST (cod. 3868) | IB, 1:12000 |
| p16^INK4a^ | pAb (rabbit) | Santa Cruz (cod. sc-467) | IB, 1:200 |
| p21 Waf1/Cip1 | 187 (mouse) | Santa Cruz (cod. sc-817) | IB, 1:200 |
| p62 | pAb (rabbit) | MBL International  (cod. PM045) | IB, 1:6000 |
| p63 | pAb (rabbit) | Abcam (cod. ab63881) | IB, 1:500 |
| Vinculin | h-VIN1(mouse) | Merck (cod. V9131) | IB, 1:1000 |

| **Secondary Antibody** | **Company and product code (cod.)** | **Application and dilution** |
| --- | --- | --- |
| Anti-rabbit IgG HRP-linked | CST (cod. 7074) | IB, 1:15000 |
| Anti-mouse IgG_k_ HRP-linked | Santa Cruz (cod. sc-516102) | IB, 1:15000 |
| Anti-rabbit IgG Alexa Fluor 555 | Thermo Fisher Scientific (cod. A-21430) | IF, 1:500 |
| Anti-mouse IgG Alexa Fluor 555 | Thermo Fisher Scientific (cod. A-11017) | IF, 1:500 |

Abbreviations: IB, Immunoblotting; IF, Immunofluorescence; CST, Cell Signaling Technology; pAb, polyclonal antibody.
